# Unique myological changes associated with ossified fabellae: a femorofabellar ligament and systematic review of the double-headed popliteus

**DOI:** 10.7717/peerj.10028

**Published:** 2020-10-16

**Authors:** Michael A. Berthaume, Spencer Barnes, Kiron K. Athwal, Lukas Willinger

**Affiliations:** 1 Division of Mechanical Engineering and Design, London South Bank University, London, UK; 2 Department of Bioengineering, Imperial College London, London, UK; 3 Department of Mechanical Engineering, Imperial College London, London, UK; 4 Department of Orthopaedic Sports Medicine, Rechts der Isar Hospital, Technical University of Munich, Munich, Germany

**Keywords:** Fabella, Myology, Double-headed popliteus, Dissection, Sesamoid bone

## Abstract

**Introduction:**

The fabella is a sesamoid bone embedded in the tendon of the lateral head of the gastrocnemius. It is the only bone in the human body to increase in prevalence in the last 100 years. As the fabella can serve as an origin/insertion for muscles, tendons, and/or ligaments (e.g., the oblique popliteal and fabellofibular ligaments), temporal changes in fabella prevalence could lead to temporal changes in “standard” knee anatomy. The aim of this study was to investigate unique myological changes to the posterolateral corner knee associated with ossified fabella presence and perform a systematic review to contextualize our results.

**Methods:**

Thirty-three fresh frozen cadaveric knees were considered. As the knees were all used for previous experimentation, the knees were in variable levels of preservation. Those with adequate preservation were used to determine ossified fabella presence/absence. When ossified fabellae were present, unique myologies associated with the fabella were recorded. A systematic review was performed on the double-headed popliteus to investigate possible correlations between this anatomical variant and the fabella.

**Results:**

Of the 33 knees, 30 preserved enough soft tissue to determine fabella presence/absence: 16/30 knees had fabellae (five cartilaginous and 11 ossified). Eight of the eleven knees with ossified fabellae retained enough soft tissue to investigate the posterolateral knee anatomy. Of these, 4/8 exhibited unique myological changes. One knee had a double-headed popliteus muscle where one head originated from the medial side of a large, bulbous fabella. A systematic review revealed double-headed popliteus muscles are rare, but individuals are 3.7 times more likely to have a fabella if they have a double-headed popliteus. Another knee had a large, thick ligament stretching from the lateral edge of the fabella to the inferoposterior edge of the lateral femoral epicondyle, deep to the lateral collateral ligament (LCL) and near the popliteal sulcus. We found no mention of such a ligament in the literature and refer to it here as the “femorofabellar ligament”. In all four knees, the plantaris and lateral gastrocnemius appeared to share a common tendinous origin, and the fabella was located at/near the junction of these muscles. In the case of the double-headed popliteus, the fabella clearly served as an origin for the plantaris.

**Conclusions:**

Despite being found in an average of 36.80% of human knees, most standard anatomical models fail to account for the fabella and/or the unique myological changes associated with fabella presence. Although our sample is small, these data highlight aspects of human biological variability generally not considered when creating generalized anatomical models. Further work is needed to identify additional changes associated with ossified fabellae and the functional consequences of omitting these changes from models.

## Introduction

The fabella is a sesamoid bone embedded in the lateral tendon of the gastrocnemius, behind the lateral femoral condyle. Although ossified fabellae are found in an average of 36.80% of knees today (as detected by dissection (Berthaume & Bull, 2020)), the fabella is often omitted from anatomical models, and is sometimes not even listed as a part of the human skeleton (Neumann & Gest, 2019; Vàsquez & Del Sol, 2020). Omission risks medical complications and inaccurate science, particularly in studies concerning biomechanics and musculoskeletal evolution. Given the fabella’s increased prevalence over the last 100 years (Berthaume, Di Federico & Bull, 2019), these consequences have increasingly significant effects.

### Exclusion of the fabella from the standard anatomical models

One of the reasons fabellae are frequently omitted from anatomical models is because of their classification as a sesamoid bone. With the exception of patellae, sesamoids are often ignored because of variation in presence, size, and location (Neumann & Gest, 2019): these reasons do not seem to apply to the fabella. Although ossified fabellae were less common in the 1800’s when critical textbooks on human anatomy were written (Berthaume & Bull, 2020; Neumann & Gest, 2019; Berthaume, Di Federico & Bull, 2019), they are now 3–4 times more common and found in an average of 36.80% of knees (Berthaume & Bull, 2020). As fabella prevalence can be found in <50% of knees in several populations, variable prevalence should not be a reason to omit fabella from anatomical models (Berthaume & Bull, 2020; Berthaume, Di Federico & Bull, 2019).

It could be argued that size variation or diminutive size are reasons for ignoring sesamoids. However, most ossified fabellae are 1–2 cm in diameter (Kojima, 1958; Chung, 1934), and diminutive size does not imply a bone is insignificant or should be overlooked. For example, the smallest of the 206 bones in the human skeleton—the stapes, malleus, and incus—are smaller than fabellae (Agathangelidis et al., 2016), but still counted as they are responsible for our ability to hear. Finally, with regards to location, while the position of some sesamoid bones may vary within the skeleton (Berthaume & Bull, in press; Corvalan, Tang & Robinson, 2018), the fabella is consistently located in the posterolateral corner of the knee (Tabira et al., 2012; Kawashima et al., 2007; Samuels, Regnault & Hutchinson, 2017), meaning variable location is not a reason to exclude the fabella from the standard anatomical model. Therefore, the three main reasons for excluding sesamoid bones (variation in presence, size, and location) do not seem to apply to the fabella (Neumann & Gest, 2019).

### Problems with excluding the fabella

Excluding the fabella from the standard anatomical model risks general ignorance of this bone in the medical and research communities. For example, the misidentification of fabellae as cyamellae in a previous study (Chen et al., 2014) has led to the suggestion that shock wave therapy can be used as a means of treatment for those with symptomatic cyamellae. Its misidentification in this study has suggested a link between cyamella presence and osteoarthritis, which is unique as the cyamella is rarely symptomatic (Berthaume & Bull, in press). Misidentification due to ignorance about the fabella may further lead to suboptimal medical treatment through clinicians being unaware of (1) problems caused by fabellae (Robertson et al., 2004; Clarke & Matthews, 1991; Heideman et al., 2011), (2) problems associated with having a fabella (Hou, 2016), (3) medical conditions associated with fabellae (Wolf & Bryk, in press; Hagihara et al., 1993; Pritchett, 1984; Ando et al., 2017); Table 1), and/or (4) long-term consequences of fabella removal (i.e., fabellectomies (Dekker et al., 2020)).

**Table 1:** Clinical issues associated with fabella presence.

Clinical issues	Condition	Source
Problems caused by the fabella	Peroneal neuropathy	Cesmebasi et al. (2016), Mangieri (1973) and Patel et al. (2013)
	Chondromalacia	Goldenberg & Wild (1952), Grisolia & Bartels (1959), and Robertson et al. (2004)
	Knee osteoarthritis	Ando et al. (2017), Hagihara et al. (1993), Pritchett (1984) and Wolf & Bryk (in press)
	Fabella-femoral osteoarthritis	Urata et al. (2015)
	Popliteal artery entrapment syndrome	Ando et al. (2017)
	Nerve palsy	Décard et al. (2017), Itoman et al. (1976), Kubota et al. (1986), Tabira et al. (2012), Takebe & Hirohata (1981)
	Rheumatoid arthritis	Uchino et al. (1992)
Pain caused by the fabella	Dislocation	Franceschi et al. (2007) and Frey et al. (1987)
	Fracture	Barreto et al. (2012), Cherrad et al. (2015), Dashefsky (1977), Heideman et al. (2011), Ikeuchi & Nagatsuka (1970), Kwee et al. (2016), Levowitz & Kletschka (1955), Marks, Cameron & Regan (1998), Sagel (1932), Tang, Mulcahy & Chew (2010), Theodorou, Theodorou & Resnick (2005), Woo (1988) and Zhou et al. (2017)
	Generalized discomfort (i.e., fabella syndrome)	Dannawi et al. (2010), Erichsen (1997), Kim et al. (2018), Rankin, Rehman & Ashcroft (2018), Segal, Miller & Krauss (2004), Seol et al. (2016), Weiner, Macnab & Turner (1977), Weiner & Macnab (1982) and Zipple, Hammer & Loubert (2003)

When fabellae become problematic, fabellectomies can be employed to relieve symptoms (Okano et al., 2016; Kuur, 1986; Wang, 1995; Kimura et al., 2019; Rankin, Rehman & Ashcroft, 2018). One study into arthroscopic fabellectomies found they were efficient at relieving symptoms associated with fabella syndrome 21+ months post-surgery, and most patients (8/10) were able to fully return to pre-operative activity levels (Dekker et al., 2020).

Exclusion of this bone has also led to a lack of research investigating the function of this bone. As such, the function of the fabella is poorly understood, and any long-term functional consequences of its removal remain unknown. Generally, the function of sesamoid bones is to relieve pressure/friction, redirect muscle lines of action, increase muscle mechanical advantage, and/or to increase tendon/ligament strength (Benjamin & Ralphs, 1998; Vogel & Koob, 1989; Shaw et al., 2008). The fabella has been hypothesized to function as a stabilizer of the posterolateral corner of the knee (Hauser et al., 2015), but it may also increase the mechanical advantage of the gastrocnemius, reducing the muscle force/energy needed for locomotion. If the fabella fulfills one or more of these functions, its removal could be detrimental, much like patellectomies (Günal & Karatosun, 2001).

Additionally, if the fabella increases the mechanical advantage of the gastrocnemius, its exclusion from biomechanical models could lead to decreased accuracy. Biomechanical models use bones, muscles, ligaments, and tendons to understand how forces are transmitted throughout the body. Biomechanical models are used for a variety of purposes—such as design of medical and recreational products, design of medical procedures, and to understand vertebrate evolution—meaning any decrease in model accuracy could lead to invalid research and results.

### Problems with including the fabella

Unfortunately, including the fabella in the standard anatomical model may not be as simple as adding an additional bone, as its presence changes the anatomy of the knee. The presence of the fabella creates an arthrodial (gliding) joint between the fabella and the lateral femoral condyle, which serves as a fourth compartment of the knee (Lencina, 2007; Zeng et al., 2012; Ehara, 2014). The articulation between the two bones can cause cartilage degeneration (Dekker et al., 2020), and/or create a “fabellar fossa” on the posterior surface of the lateral femoral condyle which stabilizes the fabella (Berthaume, Di Federico & Bull, 2019).

**Figure 1:**
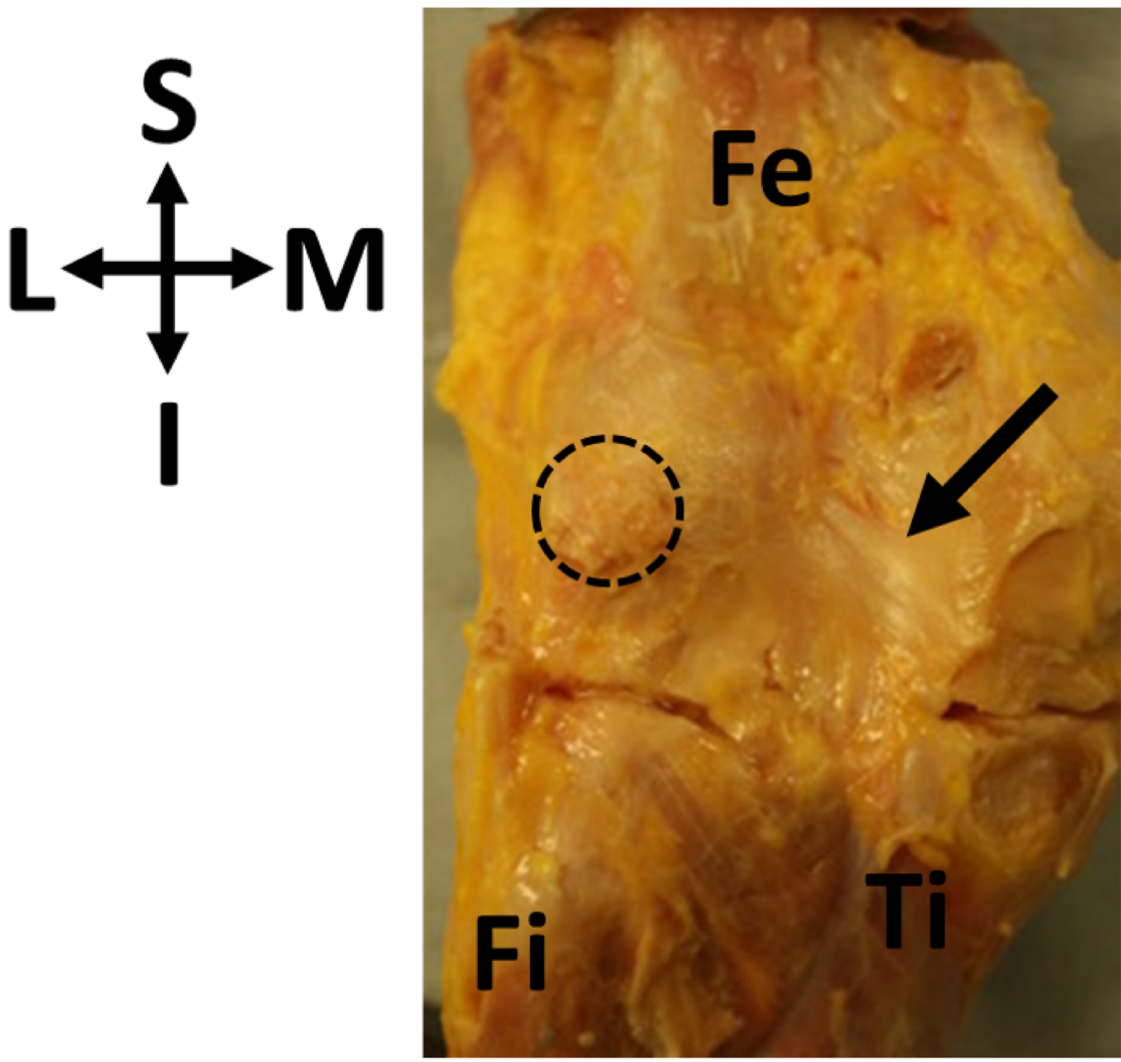
Oblique popliteal ligament (OPL, black arrow) found in association with a fabella (circle) in specimen ICL01. The knee joint capsule is dissected in this specimen, showing the border between the femur (Fe) and the tibia (Ti). Fi, fibula. Coordinate system: S, superior; I, inferior; M, medial; L, lateral.

The presence of the fabella may also lead to soft tissue changes to the posterolateral corner of the knee. The fabella is nearly always found in association with the fabellofibular ligament—which connects the distal surface of the fabella to the fibular head (Hauser et al., 2015; Minowa et al., 2004; Piyawinijwong, Sirisathira & Sricharoenvej, 2012; Driessen et al., 2014; Kurtoğlu et al., 2015)—but the ligament is not found in association with the fabella. A meta-analysis on fabellofibular ligament prevalence compiled data from over 1,000 knees and 23 studies and showed the ligament can be present in the absence of the fabella, and as such suggests the ligament be renamed the gastrocnemiofibular ligament (Pękala et al., 2019). The fabella also often serves as an attachment for the oblique popliteal ligament (OPL; also called the oblique popliteal tendon: Hedderwick et al., 2017; Fig. 1).

Other, rarer, anatomies have been found in associated with fabellae. Some studies have reported double-headed popliteus muscles where the second head originates from the fabella (Wagstaffe, 1871; Gruber, 1875). An anomalous “band” has also been observed connecting the medial surface of the fabella to the semimembranosus tendon sheath and superficial fascia of the gracilis and semitendinosus (Adukia et al., 2019). The fabella has also been reported to serve as an attachment for the fabellopopliteal ligament (Kawashima et al., 2007): however, a review of the literature revealed no dissections describing the fabellopopliteal ligament.

An improper knowledge of soft tissue anatomy of the posterolateral corner of the knee could have negative effects to clinicians and researchers alike.

Based on anatomical changes that have been reported in association with the ossified fabella, it was hypothesized that the presence of ossified fabellae would be associated with unique myological changes to the posterolateral corner of the knee in humans. We use the term “unique myological changes” to refer to changes to the muscles, tendons, or ligaments usually not associated with fabella presence: as the fabellofibular ligament and OPL are nearly always present in association with the fabella, their presence/absence is not considered a unique myological change. This will provide us with a better understanding of the variations in knee anatomy associated with ossified fabellae, which may or may not also need to be included with the fabella in the standard anatomical model.

## Materials and Methods

### The “standard” knee

To properly identify unique myological changes requires a definition of the “standard” myology of the posterolateral corner of the knee. With regards to the ossified fabella, it is embedded in the tendon of the lateral head of the gastrocnemius muscle, which wraps around lateral femoral condyle and originates on the superior surface of the condyle. The plantaris muscle originates from the same point, and there can be various levels of integration in these tendons. The medial edge of the fabella serves as an attachment for the OPL (Fig. 1), and the inferior edge of the fabella serves as an attachment for the fabellofibular ligament. Any significant deviations from this anatomy would be considered “unique anatomical changes.”

### Sample and analysis

Thirty-three fresh-frozen human cadaveric knees were procured from a tissue bank at Imperial College London. Imperial College London granted Ethical approval to carry out the study within its facilities (Ethical Application Ref: R18062). Knees were sourced from the US, and consisted of one knee/individual, usually from mid-tibial shaft to mid-femur. Dissections took place in the Biomechanics Lab in the Department of Mechanical Engineering at Imperial College London. The knees had previously been used for in vitro cadaveric experiments in the same laboratory, however posterior tissue and capsule remained fully intact in most specimens (as assessed and confirmed by a surgeon). Our sample consisted of 12 males and 21 females, ranging in age from 46 to 88 years and BMI from 17.36 to 36.91 (Table 2).

**Table 2:** Sample for this study.

Specimen ID	Age	Sex	Side	Height (cm)	Weight (kg)	BMI	Fabella	Dimensions (mm)
ICL01	54	F	L	160	58.5	22.85	Ossified	unknown
ICL02	79	M	L	175.3	57.2	18.6	No	–
ICL03	77	F	L	160	52.2	20.37	Ossified	7.4 × 4.2 × 6
ICL04	72	F	L	167.6	97.5	34.7	No	–
ICL05	72	M	L	170.2	54.9	18.95	Ossified	17.3 × 11.3 × 13.7
ICL06	71	F	L	160	44.5	17.36	No	–
ICL07	64	F	L	162.6	90.7	34.33	Ossified	11.4 × 5.8 × 11.7
ICL08	66	M	L	167.6	68	24.21	No	–
ICL09	75	M	L	165.1	91.2	33.44	No	–
ICL10	64	F	L	157.5	81.6	32.92	No	–
ICL11	74	M	L	160	56.7	22.14	Ossified	6.2 × 3.8 × 3.8
ICL12	88	F	L	170.2	71.2	24.59	Unknown	–
ICL13	84	M	L	177.8	91.6	28.98	Unknown	–
ICL14	79	F	L	157.5	45.4	18.29	Ossified	17.9 × 6.7 × 8.6
ICL15	70	F	L	165.1	99.8	36.61	Ossified	6.7 × 3.8 × 6.1
ICL16	70	F	L	157.5	54.4	21.95	Cartilaginous	–
ICL17	69	F	L	167.6	59.9	21.3	Cartilaginous	–
ICL18	79	F	L	165.1	59	21.63	Unknown	–
ICL19	70	F	L	162.6	66.2	25.06	Ossified	11.3 × 6 × 10.3
ICL20	60	F	L	172.7	65.3	21.89	Cartilaginous	–
ICL21	77	M	L	180.3	92.5	28.45	Ossified	7.4 × 3.5 × 6.5
ICL22	77	M	L	170.2	94.3	32.57	Ossified	11.8 × 8.9 × 9.8
ICL23	77	F	L	160	51.7	20.19	Ossified	9.4 × 5.1 × 12.7
ICL24	47	F	L	157.5	68	27.43	Cartilaginous	–
ICL25	61	F	L	167.6	79.4	28.24	No	–
ICL26	55	M	L	175.3	113.4	36.91	Cartilaginous	–
ICL27	57	F	L	165.1	68	24.96	No	–
ICL28	64	F	L	170.2	65.3	22.55	No	–
ICL29	46	M	L	177.8	65.8	20.8	No	–
ICL30	58	M	L	160	59	23.03	No	–
ICL31	63	F	R	167.6	70.3	25.01	No	–
ICL32	62	M	R	165.1	52.6	19.3	No	–
ICL33	62	F	L	162.6	59	22.31	No	–

**Note:**

ICL12, ICL13, and ICL18 did not have enough soft tissue preserved to determine fabella presence/absence. Height and weight are from time of death. Fabella dimensions are the maximum lengths in millimeters along the mediolateral, superoinferior, and anteroposterior axes. ICL01 was disposed of before dimensions were taken. ICL26, thought to be ossified at the time of dissection, was measured (13.5 mm × 6.5 mm × 12 mm). Specimen IDs are codenames from internal process conventions and not identifiable to patients.

When possible, the following dissection protocol was employed:

1. All skin and superficial adipose tissue around the gastrocnemius and the posterolateral corner of the knee was removed/discarded.
2. The gastrocnemius was isolated and soft tissue superficial to, but not attached to, its tendinous origins were removed.
3. Both medial and lateral heads were palpated to determine fabella presence/absence.

- If present, unique anatomies associated with the fabella were photographed and documented.
- If absent, the knee was discarded.

Myological changes that did not involve structures attached to the fabella were not investigated.

To determine if fabellae were ossified or cartilaginous, vertical incisions were made into the knee joint capsule on either side of the lateral femoral condyle, and a horizontal incision was made in the tibiofemoral joint at the height of the tibial plateau. The gastrocnemius was reflected and the fabella was lifted off the femoral condyle and palpated between the thumb and forefinger. If the fabella was thought to be non-ossified, an incision was made with a scalpel down the length of the tendon, bisecting the fabella. If this was incorrect and the fabella was ossified, this would become apparent from the scalpel contacting bone. All non-ossified fabellae were assumed to be cartilaginous, which is a safe assumption (R.F. LaPrade, 2010, personal communication). Ossified fabellae were removed from the tendon, measured, and appropriately stored for future analyses. Low-resolution micro computed tomography (microCT) was used to confirm ossification in all ossified fabellae.

Fabella presence/absence is known to be affected by parameters such as age, sex, and ethnicity (Berthaume & Bull, 2020). Due to a limited sample size, we did not investigate the relationship between any of these variables and fabella presence here. As such, no statistical analyses were performed on the data gained through dissections.

### Systematic review

During dissection, we identified an individual with a double-headed popliteus muscle, where one of the head originated from the fabella. The lead author (MAB) conducted a systematic review to summarize what is known about double-headed popliteus muscles and investigate its relation to the fabella using the following search strategies: (1) computer search of databases and (2) review of bibliographies of articles retrieved. Textbooks were not utilized unless they specifically came up in the computer search or bibliographies. This strategy is in accordance with Stroup et al. (2000). A google.scholar.co.uk was performed on 9 July 2019 using following terms:

- “accessory muscle in connection with the popliteus”
- “accessory popliteal muscle”
- “accessory popliteus”
- “double popliteus”
- “double-headed popliteus”
- “popliteal biceps”
- “popliteus biceps”
- “popliteus geminus”
- “proximal popliteal muscle”
- “proximal popliteus”
- “small popliteus”
- “supernumerary popliteal muscle”
- “supernumerary popliteus muscle”
- “three-bundle popliteus”
- “triceps popliteus”
- “triple popliteus”
- “triple-headed popliteus”
- “two-bundle popliteus”

All results were considered regardless of year of publication or language. A large variety in terms were used to accommodate the numerous names given to this condition found in the literature. For example, according to Bejjani & Jahss (1985), this muscle was called a small popliteus or proximal popliteus by Calori, popliteus biceps by Gruber, popliteus geminus by Fabrice d’Aquapendente, and accessory muscle in connection with the popliteus by Wagstaffe. Google Scholar alerts were created for search terms at the time searches were conducted to stay appraised of the literature.

Studies were selected based on the criteria that they provided information on double-headed popliteus muscles and were about humans. Where studies could not be downloaded, they were requested through interlibrary loan: if studies could not be identified through interlibrary loan, they were excluded. As most studies were case reports, risk biases were not considered and all results were lumped together for single analyses. As such, no sensitivity/subgroup analyses or meta-regressions were conducted.

## Results

Of the 33 knees, 30 had enough soft tissue preserved to investigate fabella presence/absence, in which 16 fabellae were present (53.33%). Four fabellae were confirmed to be cartilaginous during dissection. Of the fabellae believed to be ossified, 11/12 could be microCT scanned: ICL01 was disposed of before scans could be taken due to specimen management protocol. Low resolution microCT scanning revealed a bony composition in 10/11 of the fabellae. Assuming ICL01 was ossified, 11/30 fabellae were ossified (prevalence = 36.67%) and 5/30 were cartilaginous (prevalence = 16.67%).

Generally, cartilaginous fabellae appeared to be smaller than ossified ones. We could not quantify this observation as the high level of integration between the fabella and tendon made it difficult to identify the borders of the cartilaginous fabellae, and therefore take measurements. One fabella we initially thought was ossified was relatively large (ICL26 13.5 mm*6.5 mm*12 mm, Table 2), but revealed itself to be cartilaginous during low-resolution microCT scanning.

**Figure 2:**
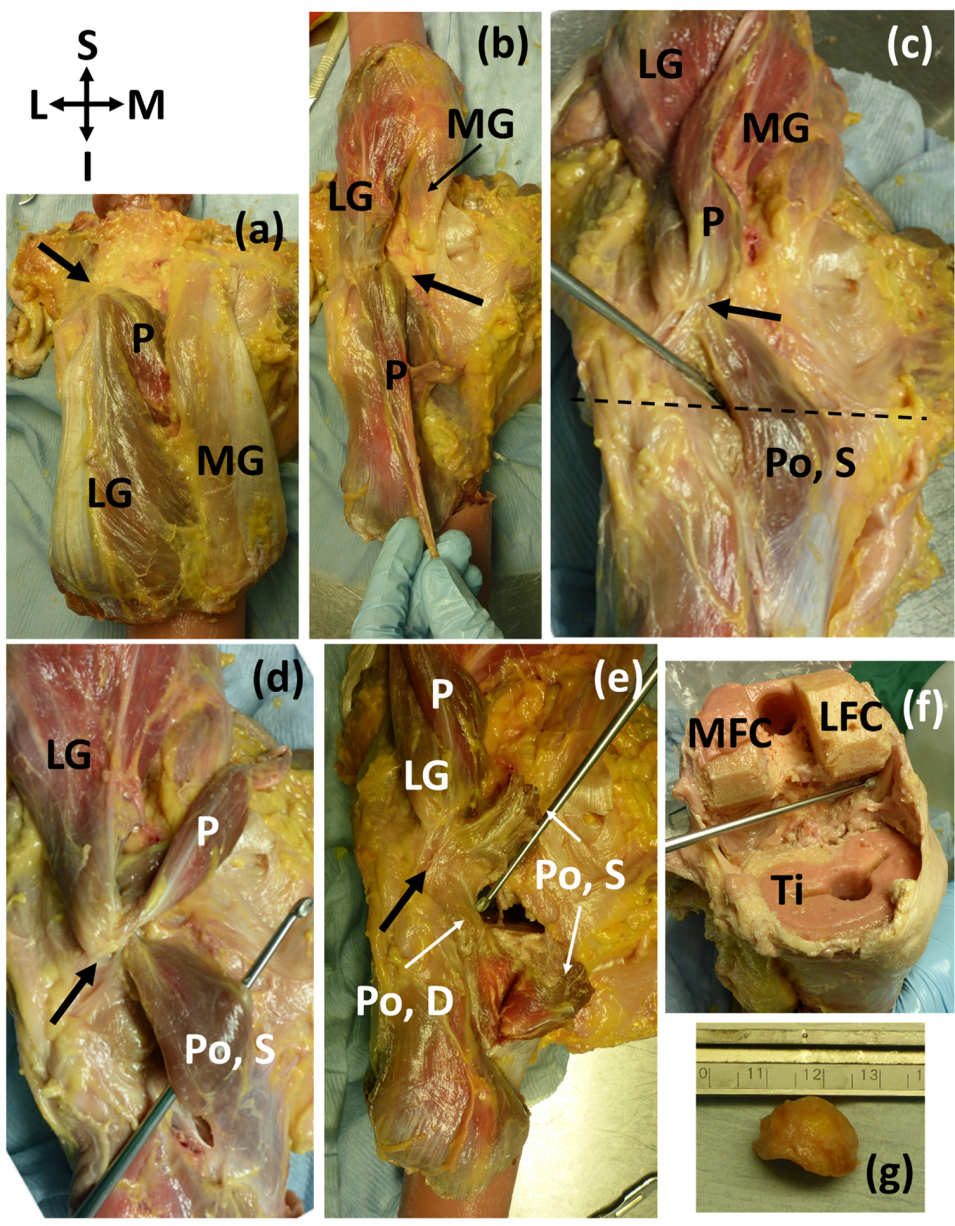
(A–E) Posterior view of specimen ICL05 with relevant coordinate system. The black arrow points towards the fabella (A) isolated medial and lateral gastrocnemii (MG, LG), with the plantaris (P) underneath (B) reflected gastrocnemii showing the plantaris (P) (C) reflected plantaris showing the superior popliteal head (Po, S). Dotted line represents the tibial plateau (D) image showing how the superior head of the popliteus, plantaris, and lateral head of the gastrocnemius converge into the fabella before originating from the femur from one, common tendon (E) the superior head of the popliteus was bisected, revealing the deep head of the popliteus (Po, D) (F) the distal end of the femur and proximal end of the tibia (Ti) were removed for a previous experiment. The patella and surrounding tissue were removed to view the knee joint from the anterior side, and the knee was bent, revealing the popliteal tendon at the end of the dissecting probe, which connected to the deep head of the popliteus. The pink substance is bone cement (from previous experiments). MFC = medial femoral condyle, LFC = lateral femoral condyle (G) lateral view of the large, bulbous fabella. The lower, flatter side articulated with the femur. The fabella was nearly a perfect hemispherical dome.

**Figure 3:**
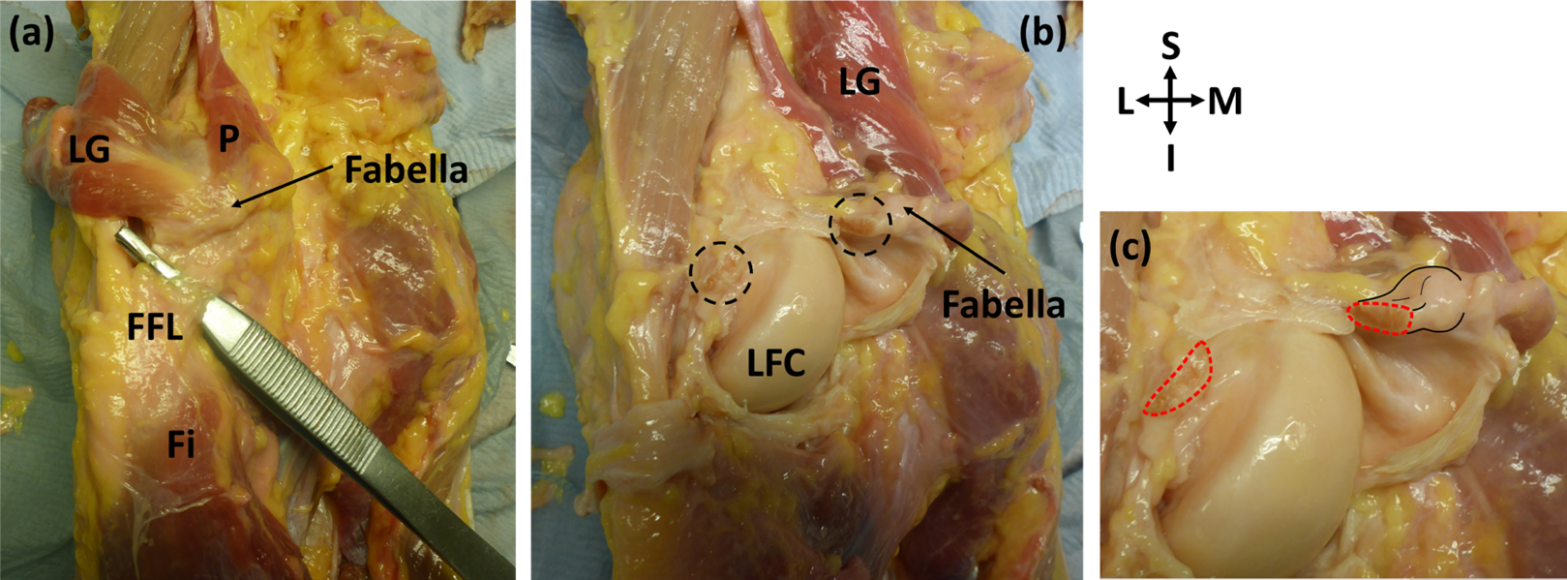
Posterior view of specimen ICL23. (A) The lateral gastrocnemius (LG) has been separated from the medial gastrocnemius and reflected with the plantaris (p). Note how the plantaris is originating from the tendon of the lateral head of the gastrocnemius, and the fabella is located at the intersection of these two muscles. The fabellofibular ligament (FFL) is connecting the fabella to the fibula (Fi) (B) the knee capsule has been cut into and reflected to view the underside of the fabella. The cross-sections of the bisected large, thick ligament can be seen in the dotted circles. LFC = lateral femoral condyle (C) close up of bisected ligament. Cross-sections highlighted with red dotted line. Attachment of the ligament to the fabella and the fabella itself are highlighted with black lines.

Of the 11 knees with ossified fabellae, ICL01, ICL 14, and ICL19 lacked enough soft tissue to confidently investigate unique myological changes (e.g., Fig. 1). Of the 8 remaining knees, 4 exhibited unique changes: ICL05 had a double-headed popliteus where the second head originated from a large, bulbous fabella (Fig. 2), ICL23 had a large, thick ligament stretching from the lateral edge of the fabella to the inferoposterior edge of the lateral femoral epicondyle, near the popliteal sulcus (Fig. 3). Finally, the plantaris and gastrocnemius appeared to share a common tendinous origin in ICL03, ICL05, ICL15, and ICL23. In ICL05, the fabella clearly served as an origin not just for the second popliteal head, but also the plantaris (Fig. 2). ICL07, ICL11, ICL21, and ICL22 did not exhibit any unique myological differences from standard anatomy.

### Systematic review

**Figure 4:**
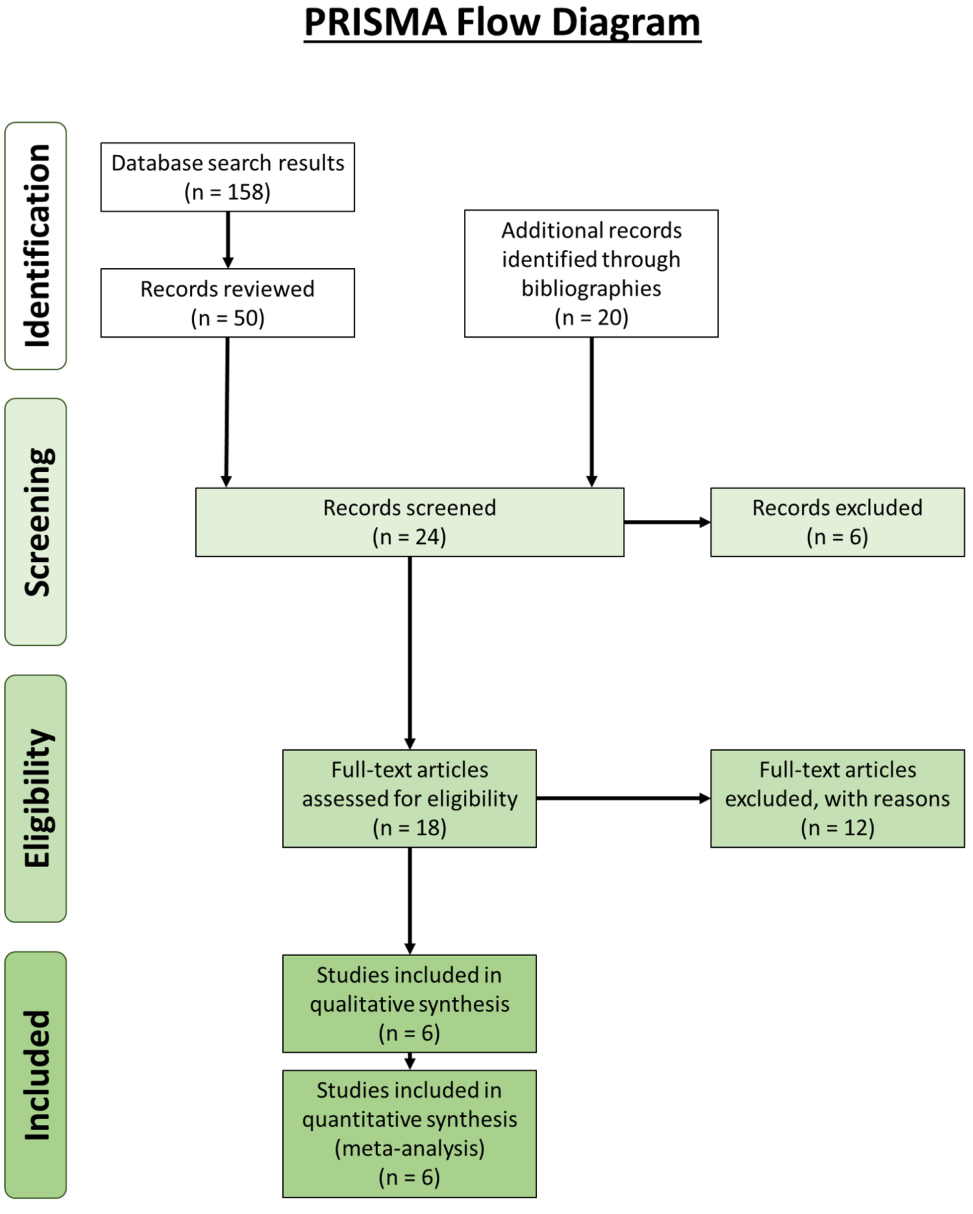
Preferred Reporting Items for Systematic Reviews and Meta-Analyses (PRISMA) flow diagram (Moher et al., 2009) for the double-headed popliteus systematic review.

Our review revealed 158 unique results, of which 50 records were reviewed (Fig. 4). An additional 20 records were identified through bibliographic reviews. Of the 70 results, 24 were screened further. Six records identified through bibliographic review were excluded because we could not locate usable copies: Calori (1866), Bevan (year unknown), Riolan (year unknown) and Fabrice d’Aquapendente (1687) from Bejjani & Jahss (1985), and Testut (1884) and Nordlund et al., (1877) from Fürst (1903). Of the remaining 18 records, 12 were excluded: seven contained no original data, two were about supernumerary muscle bundles of the popliteus/accessory popliteal muscles and not double-headed popliteus muscles (Duc et al., 2004; Hahn et al., 2019), and 3 were about proximal popliteal attachments but did not encounter any double-headed popliteus muscles (Fürst, 1903; Upasna et al., 2016; Feipel, Simonnet & Rooze, 2003). It is not uncommon for the origin of the popliteus to have two or more attachments, but these are not separate heads (Upasna et al., 2016). The results from the remaining six are presented in Table 3.

**Table 3:** Available studies providing primary data on double-headed popliteus muscles.

Num. of knees	Num. of ind.	Num. of double-headed popliteus muscles	Age (years)	Sex	Bi- or uni-lateral	Fabella	Origin of superior/medial head	Insertion of superior/medial head	Detection method	References
2	1	2	65	M	Bilateral	NA	lateral condyle of the femur	popliteus muscle	Dissection	Kurtoğlu et al. (2015)
2 or 4	NA	2 or 4	70, 22	M, M	NA	yes, no	(1) aponeurotic band from the fabella; (2) near the origin of the gastrocnemius, attached to the knee joint capsule by a few rare fibers	(1) popliteus muscle (2) oblique line on the posterior aspect of the tibia, where it blended with the popliteus	Dissection	Gruber (1875)
40	NA	1	NA	NA	NA	NA	NA	NA	Dissection	Bartoníček (2005)
500	250	11	one: 21, ten others: NA	8 M	3 bilateral, 5 unilateral (3R, 2L)	7 yes, 4 no	the fabella, when present, otherwise from the femur or the posterior ligament of the knee joint	fuzes with the popliteus muscle, sits against the triangular field above the popliteal line of the posterior surface of the tibia	Dissection	Piyawinijwong, Sirisathira & Sricharoenvej (2012)
1039	NA	3	NA	NA	NA	NA	NA	NA	MRI	Koplas et al. (2009)
1	1	1	NA	NA	NA, found in L leg	yes	Inner side of the fabella	inner edge of the tibia as far as the oblique line, and also blending with the fibers of the popliteus	Dissection	Minowa et al. (2004)
30	30	1	72	M	NA, found in L leg	yes	Medial side of the fabella	fuzes with the popliteus muscle before inserting into the posterior surface of the tibia, proximal to the soleus	Dissection	This study

**Note:**

Interestingly, the plantaris was missing from Minowa et al. (2004) and from 2/11 individuals with double-headed popliteus muscles in Piyawinijwong, Sirisathira & Sricharoenvej (2012). In addition, (Piyawinijwong, Sirisathira & Sricharoenvej, 2012, Gruber, 1875) each reported a case where the popliteal artery passed between the two popliteal heads: the position of the popliteal artery relative to the popliteus was not examined in this study. For the bi/unilateral column, information on the contralateral leg was not available in two cases (Minowa et al., 2004 and this study). In the case of Gruber (1875), it was not possible to tell if one or both knees per individual were examined. The skew towards males in Piyawinijwong, Sirisathira & Sricharoenvej (2012) is due to the heavily skewed sample, which was mostly male (*n* = 242 M, 8 F). The age was only available for 1/11 individuals in Piyawinijwong, Sirisathira & Sricharoenvej (2012) : the other 10 were beggars. Finally, the popliteus was not being rigorously examined in Koplas et al. (2009), and the three cases were found incidentally. It is possible the incidence rate is higher for this study.

**Figure 5:**
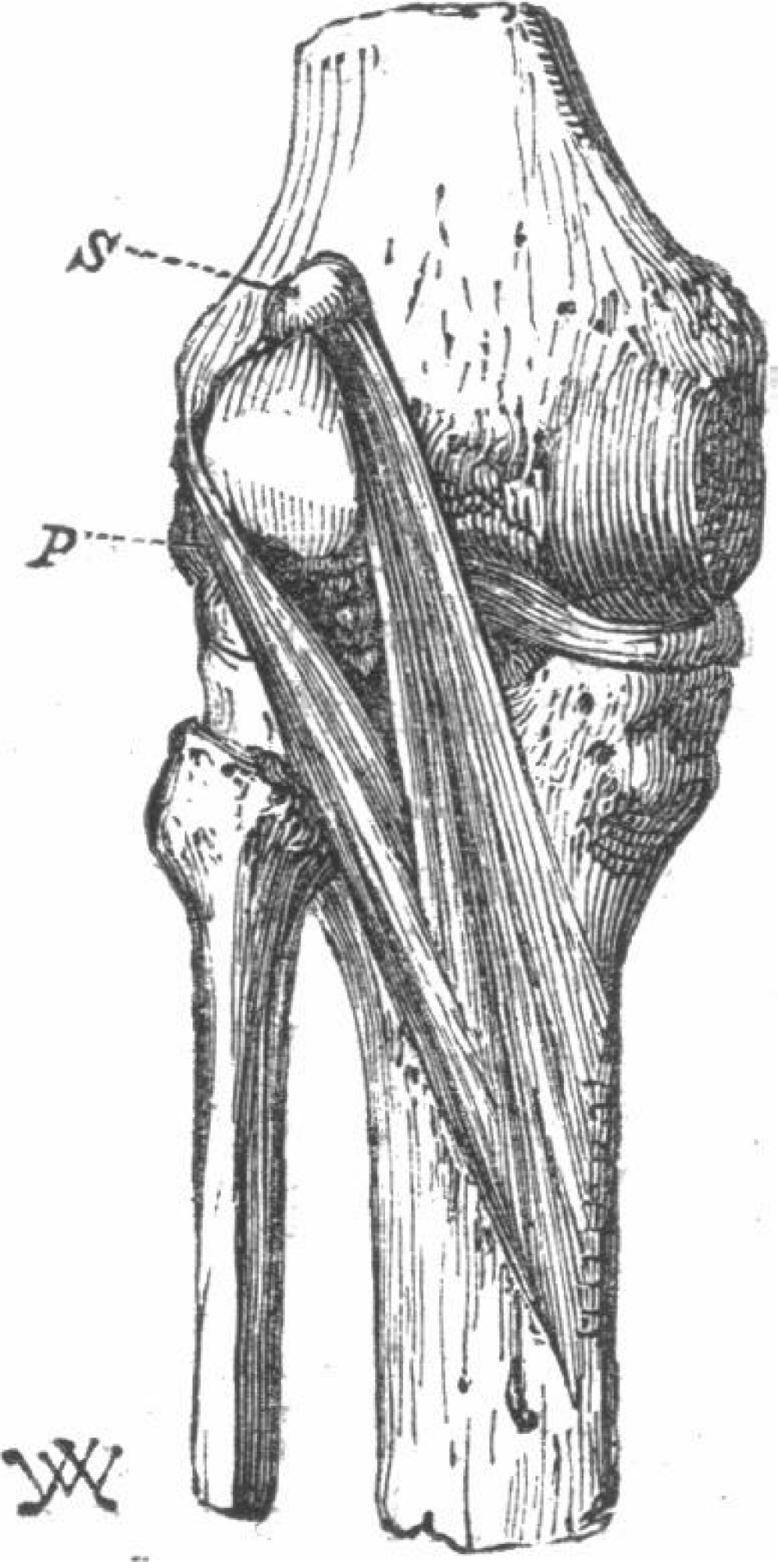
The first reported double-headed popliteus in humans (Wagstaffe, 1871).

The first study reporting on the double-headed popliteus was from 1871 (Wagstaffe, 1871). Like ours, the superior head of their popliteus originated from a large fabella, but unlike ours, the plantaris was missing from this individual (Fig. 5). Following the publication of (Wagstaffe, 1871), Gruber reviewed his dissection notes and, of the 250 cadavers considered (*n* = 242 M, 8 F) and found 11 cases of double-headed popliteus muscles, all in men (prevalence = 4.4%). Three of the cases were bilateral and 5 unilateral. Of the unilateral cases, 3 were found in right legs, 2 in left, and most individuals with double-headed popliteus muscles also had plantaris muscles (9/11). Gruber proposed two classes of double-headed popliteus muscles, one in which the two heads were roughly the same size, and one in which the deep, lateral head was bigger. The double-headed popliteus identified in this study does not fit in either classification, as the superior, medial head was larger.

The last four studies from the systematic review more-or-less discuss the double-headed popliteus in passing. One study reviewing lower limb muscular anatomy mentioned two men with double-headed popliteus muscles (Bejjani & Jahss, 1985): one man was 70 years old and had fabella(e), and the other was 22 years old and did not. Another study mentioned finding a double-headed popliteus while dissecting 40 knees but gave no further information (Fabbriciani & Oransky, 1992). Two double-headed popliteus muscles (one per leg) were found during dissection of a 65 year old male (Bartoníček, 2005), and 3 were incidentally found when reviewing 1039 MRI scans of legs looking for triple-headed gastrocnemii (Koplas et al., 2009). As the last study was not explicitly looking for double-headed popliteus muscles, it is possible their prevalence rate of 0.3% is a minimum prevalence rate. We combined the data from the systematic review with our results for the results presented below.

Fabellae were present in 63.6% (7/11) of Gruber’s cases. At a similar time, Gruber published a fabella prevalence rate study (Gruber, 1875; Hessen, 1946), where he found fabella prevalence to be 17.1% (400/2340). Assuming none of these individuals had double-headed popliteal muscles^1^ 1There is no way of knowing how many of the individuals in this sample had double-headed popliteus muscles. However, if a person is more likely to have a fabella when they have a double-headed popliteus muscle, the inclusion of individuals with double-headed popliteus muscles would only serve to increase fabella prevalence in this population, decreasing the chances of finding a difference in prevalence between our two groups., fabellae are ~3.7 times more common in individuals with double-headed popliteus muscles (χ-squared = 16.568, simulated *p*-value = 0.0013 (RStudio Team, 2016; R Core Team, 2018)).

While rare, double-headed popliteus muscle prevalence rates range from 0.3% to 4.4%. Of the 12 individuals who had sex reported, all were male (Table 3), possibly due to sampling bias. Although the sex distribution from Bejjani & Jahss (1985) and Bartoníček (2005) were not known, the sample from Gruber (1875) was predominantly male and our sample was predominantly female. Bilateral cases (4) were as common as unilateral ones (5), and the youngest individual known to have a double-headed popliteus was 21. Interestingly, the popliteal artery can pass between the two heads (Gruber, 1875; Bejjani & Jahss, 1985), although we did not observe this in our individual.

Four studies reported on fabella presence/absence in association with the double-headed popliteus (Wagstaffe, 1871; Gruber, 1875; Bejjani & Jahss, 1985), and fabellae were present in 10/15 of the cases. Given lack of data, we cannot conclude whether double-headed popliteus muscles are more/less common when fabellae are present, but these results imply fabellae are more common in knees with double-headed popliteus muscles. Finally, 3 cases also reported a lack of plantaris muscle when the double-headed popliteus was present.

## Discussion

The results from our dissections support our hypothesis that the presence of ossified fabellae is associated with unique myological changes to posterolateral corner of the knee. Due to our modest sample size, any conclusions about the inclusion of these changes in the standard anatomical model cannot be made. However, half of the knees with ossified fabellae examined further (4/8) exhibited unique myological changes, suggesting unique changes may not be uncommon when ossified fabellae are present, and more studies with larger samples are needed to investigate this question, particularly as ossified fabellae become increasingly common.

In all four knees that possessed unique myological changes, the origin of the plantaris shifted from the femur’s lateral supracondylar ridge to the tendon of the lateral head of the gastrocnemius (Fig. 2), implying contraction of either the gastrocnemius or the plantaris could cause the tendon to become stressed. In these cases, the fabella was located at/near the junction of these muscles, where it may have been strengthening the connection between the plantaris and gastrocnemius. Two out of these four knees exhibited further unique myological changes: one had a double-headed popliteus, and one had a unique ligament (herein, the femorofabellar ligament). Here, we discuss the results of these unique myological changes in further detail, and present hypotheses about what these results imply concerning the function of the fabella.

### Double-headed popliteus muscle

The large, bulbous fabella in ICL05 served as an origin for both the plantaris and a double-headed popliteus muscle (Fig. 2). The popliteus muscle can have multiple origins, such as an aponeuroses attaching popliteal fibers to the meniscus, popliteal muscle fibers attaching to the knee capsule above the lateral meniscus, and/or the popliteal tendon fuzing with the arcuate ligament^2^ 2The attachment to the arcuate ligament has also been described as a condensation of fibers coming from the popliteal head to the fibula, and not as an attachment to the arcuate. (Bartoníček, 2005; Last, 1948, 1950). However, these cases are different from what we observed, where the two head of the popliteus were distinct and separate.

The double-headed popliteus in ICL05 consisted of a larger, superficial head that originated from the fabella and a smaller, deep head that originated from the lateral femoral epicondyle via the popliteal tendon. The two heads were separable near their origins but fuzed before inserting into the posterior surface of the tibia, proximal to the medial body of the soleus. We investigated the level of integration by attempting to separate the two heads: this caused the fibers past the point of integration becoming shredded (i.e., the bright red fibers in Fig. 2E).

Our systematic review revealed double-headed popliteus muscles are rare but can have prevalence rates up to 4.4%. Using Gruber’s data, it appears fabellae are ~3.7 times more common when a double-headed popliteus is present, suggesting there may be a link between the two anatomical anomalies.

### Femorofabellar ligament

The femorofabellar ligament in ICL23 was deep, short, and not immediately visible upon dissection. It connected the lateral side of the fabella to the inferoposterior edge of the lateral femoral epicondyle, near the popliteal sulcus (Fig. 3), and ran deep to the lateral collateral ligament (LCL). It was discovered when the vertical incisions were made into the knee capsule, as it was extremely difficult to bisect. Given its location, we could not obtain clear pictures while the ligament was intact. It was bisected as close to its femoral attachment as possible, and the portion of the ligament attached to the fabella was <1 cm in length (Fig. 3).

Reviewing the literature, we failed to identify any similar ligament in humans. The attachment site of this ligament to the fabella is similar to that of the femoropatellar ligament observed in the stifle joint in some mammals, such as canines and felids. However, the ligament we observed is not the femoropatellar ligament, as the femoropatellar ligament bypasses the femur, connecting the patella to the fabella (Carpenter & Cooper, 2000). Given the ligament’s origin and insertion, the authors have termed it the “femorofabellar ligament.”

### Functional implications of the ossified fabella

Few studies have investigated fabella function. One found the fabellofibular ligament was larger/more robust when the fabella was present, and suggested the fabella may induce ligament development (Minowa et al., 2004), helping reinforce and stabilize the posterolateral corner of the knee (Hauser et al., 2015; Minowa et al., 2004; Eyal et al., 2019). Other studies have suggested the fabella may be acting like a patella, increasing the mechanical advantage of the muscle in which it is embedded (Zeng et al., 2012; Driessen et al., 2014; Mottershead, 1988).

One function that is not discussed is the role of the fabella in strengthening the connection between the muscles, ligaments, and tendons. If true, this would suggest larger, bony fabellae form when they are exposed to higher levels of mechanical stimulation: this function is supported by multiple lines of evidence. First, the relationship between fabella and fabellofibular ligament size supports the idea of coincident growth and development between structures. As the fabellofibular ligament can be found when the fabella is absent but the fabella cannot be found if the fabellofibular ligament is absent (Kaplan, 1961), it is more likely that the fabellofibular ligament is inducing fabellar growth and development than the other way around (Pękala et al., 2019). This would suggest the fabella could be strengthening the connection between the fabellofibular ligament and the gastrocnemius (Shaw et al., 2008). Second, in our study, ossified fabellae were often found at the intersection of the plantaris and gastrocnemius, suggesting the fabella may be strengthening this connection. And finally, fabellae often serve as an origin for double-headed popliteus muscles (when present), suggesting they may be strengthening the connection between this muscle and the gastrocnemius tendon. However, this is likely not the fabella’s only function, fabellofibular ligaments are found fabellae are absent, and there are not always extra muscle bundles originating from the gastrocnemius when the fabella is present.

Ultimately, the fabella is likely a multifunctional bone, fulfilling more than one function.

### Ossified fabellae and the standard anatomical model

It is clear there are several myological changes commonly found in association with the ossified fabella (i.e., the fabellofibular ligament and OPL): should the fabella be included in the standard anatomical model, we recommend these should be included as well. There are several less common myological changes that have been documented. While we do not recommend these changes be included in the standard anatomical model, they should be documented and shared with the clinical and scientific community. While some of these human biological variations may be insignificant, others may have significant effects in clinical practice and research.

## Conclusions

Most standard anatomical models fail to account for the fabella and/or the unique myological differences from standard anatomy associated with fabella presence. Here, we show how unique myological changes associated with the ossified fabella that can have significant anatomical and biomechanical considerations are not uncommon. These myological changes suggest one of the fabella’s functions may be to strengthen the connection between the muscles, ligaments, and tendons of the posterolateral corner of the knee.

Although our sample is small, these data highlight aspects of human biological variability generally not considered when creating generalized anatomical models. Further work is needed to identify additional changes associated with ossified fabellae and the functional consequences of omitting these changes from models.

## Supplemental Information

10.7717/peerj.10028/supp-1 Supplemental Information 1 Rationale for systematic review/meta-analysis.

10.7717/peerj.10028/supp-2 Supplemental Information 2 PRISMA checklist.

## Additional Information and Declarations

### Data Availability

The following information was supplied regarding data availability: All raw data are available in the tables in the article.
