# Supplementary material for: Unique myological changes associated with ossified fabellae: a femorofabellar ligament and systematic review of the double-headed popliteus
Source: PeerJ. 2020 Oct 16;8:e10028. doi: 10.7717/peerj.10028 (PMC7571414; doi:10.7717/peerj.10028)
Supplement: Supplemental Information 1 [file peerj-08-10028-s001.docx]

**Systematic Review and/or Meta-Analysis Rationale**

For systematic reviews / meta-analyses, authors are asked to provide the following information:

1. The rationale for conducting the meta-analysis;
2. The contribution that the meta-analysis makes to knowledge in light of previously published related reports, including other meta-analyses and systematic reviews.

Please upload your answers in a supplemental file (File type 'Other').

**Rationale**

The aim of this paper was to document unique myological changes to the posterolateral corner of the knee that occur in association with ossified fabellae. During dissection, we identified an individual with a double-headed popliteus where the larger head originated from a large, bulbous fabella: such a unique anatomy has major implications to the anatomical and biomechanical communities. As none of the authors were previously aware of double-headed popliteus muscles in humans, or of any muscles originating from fabellae, we questioned whether there was a relationship between fabella presence and double-headed popliteus muscles.

Upon reviewing the literature, the first paper we found on the topic was Wagstaffe, 1875, who reported a dissection that yielded results nearly identical to ours. Given the paucity of literature on the topic, we felt it prudent to perform a systematic review to gather all published material on double-headed popliteus muscles and investigate the possible relationship between the fabella and double-headed popliteus muscles.

**Contribution**

Unfortunately, given the rarity of this muscle in the literature and the fact that little more is reported on the muscle past presence/absence, there was little we could do in terms of quantitative analyses on the topic. This is not typical of a meta-analysis, which usually involve some sort of quantitative analysis, but felt what we did was still a meta-analysis as we examined data from independent studies to determine overall trends. We qualitatively summarized what data was available and showed that, when double-headed popliteus muscles are present, fabellae are often also present. The potential relationship between these two structures indicates one manner in which musculoskeletal anatomical flexibility can be achieved in a conservative bauplan.
