# Supplementary material for: Unique myological changes associated with ossified fabellae: a femorofabellar ligament and systematic review of the double-headed popliteus
Source: PeerJ. 2020 Oct 16;8:e10028. doi: 10.7717/peerj.10028 (PMC7571414; doi:10.7717/peerj.10028)
Supplement: Supplemental Information 2 [file peerj-08-10028-s002.docx]

| **Section/topic** | **#** | **Checklist item** | **Reported on page #** |
| --- | --- | --- | --- |
| **TITLE** | | |  |
| Title | 1 | Identify the report as a systematic review, meta-analysis, or both.  Unique myological changes associated with ossified fabellae: a femorofabellar ligament and systematic review of the double-headed popliteus | 1 |
| **ABSTRACT** | | |  |
| Structured summary | 2 | Provide a structured summary including, as applicable: background; objectives; data sources; study eligibility criteria, participants, and interventions; study appraisal and synthesis methods; results; limitations; conclusions and implications of key findings; systematic review registration number.  Due to space limitations, fewer words could be dedicated to the systematic review in our abstract than we would have liked. See text below:  A systematic review was performed on the double-headed popliteus to investigate possible correlations between this anatomical variant and the fabella.  A systematic review revealed double-headed popliteus muscles are rare, but individuals are 3.7 times more likely to have a fabella if they have a double-headed popliteus. | 2 |
| **INTRODUCTION** | | |  |
| Rationale | 3 | Describe the rationale for the review in the context of what is already known.  The systematic review is not specifically discussed until the Material and Methods, as it was conducted in response to initial findings of the study, when we found a unique myological change in association with a fabella. As such, the following paragraph in the Introduction is the most relevant to the systematic review here.  Based on anatomical changes that have been reported in association with the ossified fabella, it was hypothesized that the presence of ossified fabellae would be associated with unique myological changes to the posterolateral corner of the knee in humans. We use the term “unique myological changes” to refer to changes to the muscles, tendons, or ligaments usually not associated with fabella presence: as the fabellofibular ligament and OPL are nearly always present in association with the fabella, their presence/absence is not considered a unique myological change. This will provide us with a better understanding of the variations in knee anatomy associated with ossified fabellae, which may or may not also need to be included with the fabella in the standard anatomical model. | 5 |
| Objectives | 4 | Provide an explicit statement of questions being addressed with reference to participants, interventions, comparisons, outcomes, and study design (PICOS).  Based on anatomical changes that have been reported in association with the ossified fabella, it was hypothesized that the presence of ossified fabellae would be associated with unique myological changes to the posterolateral corner of the knee in humans. We use the term “unique myological changes” to refer to changes to the muscles, tendons, or ligaments usually not associated with fabella presence: as the fabellofibular ligament and OPL are nearly always present in association with the fabella, their presence/absence is not considered a unique myological change. This will provide us with a better understanding of the variations in knee anatomy associated with ossified fabellae, which may or may not also need to be included with the fabella in the standard anatomical model. | 5 |
| **METHODS** | | |  |
| Protocol and registration | 5 | Indicate if a review protocol exists, if and where it can be accessed (e.g., Web address), and, if available, provide registration information including registration number.  The lead author (MAB) conducted a systematic review to summarize what is known about double-headed popliteus muscles and investigate its relation to the fabella using the following search strategies: (1) computer search of databases and (2) review of bibliographies of articles retrieved. Textbooks were not utilized unless they specifically came up in the computer search or bibliographies. This strategy is in accordance with [45]. A google.scholar.co.uk was performed on 9 July 2019 using following terms:  • "accessory muscle in connection with the popliteus"  • "accessory popliteal muscle"  • "accessory popliteus"  • "double popliteus"  • "double-headed popliteus"  • "popliteal biceps"  • "popliteus biceps"  • "popliteus geminus"  • "proximal popliteal muscle"  • "proximal popliteus"  • "small popliteus"  • "supernumerary popliteal muscle"  • "supernumerary popliteus muscle"  • "three-bundle popliteus"  • "triceps popliteus"  • "triple popliteus"  • "triple-headed popliteus"  • "two-bundle popliteus"  …A large variety of terms were used to accommodate the numerous names given to this condition found in the literature. For example, according to [46], this muscle was called a small popliteus or proximal popliteus by Calori, popliteus biceps by Gruber, popliteus geminus by Fabrice d’Aquapendente, and accessory muscle in connection with the popliteus by Wagstaffe. Google Scholar alerts were created for search terms at the time searches were conducted to stay appraised of the literature. | NA |
| Eligibility criteria | 6 | Specify study characteristics (e.g., PICOS, length of follow-up) and report characteristics (e.g., years considered, language, publication status) used as criteria for eligibility, giving rationale.  All results were considered regardless of year of publication or language… Studies were selected based on the criteria that they provided information on double-headed popliteus muscles and were about humans. Where studies could not be downloaded, they were requested through interlibrary loan: if studies could not be identified through interlibrary loan, they were excluded. As most studies were case reports, risk biases were not considered and all results were lumped together for single analyses. As such, no sensitivity/subgroup analyses or meta-regressions were conducted. | 6 |
| Information sources | 7 | Describe all information sources (e.g., databases with dates of coverage, contact with study authors to identify additional studies) in the search and date last searched.  The lead author (MAB) conducted a systematic review to summarize what is known about double-headed popliteus muscles and investigate its relation to the fabella using the following search strategies: (1) computer search of databases and (2) review of bibliographies of articles retrieved. Textbooks were not utilized unless they specifically came up in the computer search or bibliographies. This strategy is in accordance with [45]. A google.scholar.co.uk was performed on 9 July 2019 using following terms: | 5 |
| Search | 8 | Present full electronic search strategy for at least one database, including any limits used, such that it could be repeated.  All results were considered regardless of year of publication or language… Studies were selected based on the criteria that they provided information on double-headed popliteus muscles and were about humans. Where studies could not be downloaded, they were requested through interlibrary loan: if studies could not be identified through interlibrary loan, they were excluded. As most studies were case reports, risk biases were not considered and all results were lumped together for single analyses. As such, no sensitivity/subgroup analyses or meta-regressions were conducted. | 5-6 |
| Study selection | 9 | State the process for selecting studies (i.e., screening, eligibility, included in systematic review, and, if applicable, included in the meta-analysis).  Studies were selected based on the criteria that they provided information on double-headed popliteus muscles and were about humans.  The section “Systematic review” In the results section provides study-by-study explanations for which studies were included/excluded. | 6, 7-8 |
| Data collection process | 10 | Describe method of data extraction from reports (e.g., piloted forms, independently, in duplicate) and any processes for obtaining and confirming data from investigators.  All possible data was extracted, although this is not explicitly stated. The following paragraph depicts what was done.  Studies were selected based on the criteria that they provided information on double-headed popliteus muscles and were about humans. Where studies could not be downloaded, they were requested through interlibrary loan: if studies could not be identified through interlibrary loan, they were excluded. As most studies were case reports, risk biases were not considered and all results were lumped together for single analyses. As such, no sensitivity/subgroup analyses or meta-regressions were conducted. | 6 |
| Data items | 11 | List and define all variables for which data were sought (e.g., PICOS, funding sources) and any assumptions and simplifications made. | NA |
| Risk of bias in individual studies | 12 | Describe methods used for assessing risk of bias of individual studies (including specification of whether this was done at the study or outcome level), and how this information is to be used in any data synthesis. | NA |
| Summary measures | 13 | State the principal summary measures (e.g., risk ratio, difference in means).  This information is given throughout the Results and Discussion, but mostly presented in the following paragraphs of the Results section  We combined the data from the systematic review with our results for the results presented below.  Fabellae were present in 63.6% (7/11) of Gruber’s cases. At a similar time, Gruber published a fabella prevalence rate study [55,56], where he found fabella prevalence to be 17.1% (400/2340). Assuming none of these individuals had double-headed popliteal muscles , fabellae are ~3.7 times more common in individuals with double-headed popliteus muscles (χ-squared = 16.568, simulated p-value = 0.0013 [57,58]).  While rare, double-headed popliteus muscle prevalence rates range from 0.3-4.4%. Of the 12 individuals who had sex reported, all were male (Table 3), possibly due to sampling bias. Although the sex distribution from [46,53] were not known, the sample from [43] was predominantly male and our sample was predominantly female. Bilateral cases (4) were as common as unilateral ones (5), and the youngest individual known to have a double-headed popliteus was 21. Interestingly, the popliteal artery can pass between the two heads [43,46], although we did not observe this in our individual.  Four studies reported on fabella presence/absence in association with the double-headed popliteus [42,43,46], and fabellae were present in 10/15 of the cases. Given lack of data, we cannot conclude whether double-headed popliteus muscles are more/less common when fabellae are present, but these results imply fabellae are more common in knees with double-headed popliteus muscles. Finally, 3 cases also reported a lack of plantaris muscle when the double-headed popliteus was present. | 8-9 |
| Synthesis of results | 14 | Describe the methods of handling data and combining results of studies, if done, including measures of consistency (e.g., I^2^) for each meta-analysis. | NA |

Page 1 of 2

| **Section/topic** | **#** | **Checklist item** | **Reported on page #** |
| --- | --- | --- | --- |
| Risk of bias across studies | 15 | Specify any assessment of risk of bias that may affect the cumulative evidence (e.g., publication bias, selective reporting within studies). | NA |
| Additional analyses | 16 | Describe methods of additional analyses (e.g., sensitivity or subgroup analyses, meta-regression), if done, indicating which were pre-specified. | NA |
| **RESULTS** | | |  |
| Study selection | 17 | Give numbers of studies screened, assessed for eligibility, and included in the review, with reasons for exclusions at each stage, ideally with a flow diagram.  Figure 4 | 8 |
| Study characteristics | 18 | For each study, present characteristics for which data were extracted (e.g., study size, PICOS, follow-up period) and provide the citations.  We combined the data from the systematic review with our results for the results presented below.  Fabellae were present in 63.6% (7/11) of Gruber’s cases. At a similar time, Gruber published a fabella prevalence rate study [55,56], where he found fabella prevalence to be 17.1% (400/2340). Assuming none of these individuals had double-headed popliteal muscles , fabellae are ~3.7 times more common in individuals with double-headed popliteus muscles (χ-squared = 16.568, simulated p-value = 0.0013 [57,58]).  While rare, double-headed popliteus muscle prevalence rates range from 0.3-4.4%. Of the 12 individuals who had sex reported, all were male (Table 3), possibly due to sampling bias. Although the sex distribution from [46,53] were not known, the sample from [43] was predominantly male and our sample was predominantly female. Bilateral cases (4) were as common as unilateral ones (5), and the youngest individual known to have a double-headed popliteus was 21. Interestingly, the popliteal artery can pass between the two heads [43,46], although we did not observe this in our individual.  Four studies reported on fabella presence/absence in association with the double-headed popliteus [42,43,46], and fabellae were present in 10/15 of the cases. Given lack of data, we cannot conclude whether double-headed popliteus muscles are more/less common when fabellae are present, but these results imply fabellae are more common in knees with double-headed popliteus muscles. Finally, 3 cases also reported a lack of plantaris muscle when the double-headed popliteus was present. | 8 |
| Risk of bias within studies | 19 | Present data on risk of bias of each study and, if available, any outcome level assessment (see item 12). | NA |
| Results of individual studies | 20 | For all outcomes considered (benefits or harms), present, for each study: (a) simple summary data for each intervention group (b) effect estimates and confidence intervals, ideally with a forest plot. | NA |
| Synthesis of results | 21 | Present results of each meta-analysis done, including confidence intervals and measures of consistency.  We combined the data from the systematic review with our results for the results presented below.  Fabellae were present in 63.6% (7/11) of Gruber’s cases. At a similar time, Gruber published a fabella prevalence rate study [55,56], where he found fabella prevalence to be 17.1% (400/2340). Assuming none of these individuals had double-headed popliteal muscles , fabellae are ~3.7 times more common in individuals with double-headed popliteus muscles (χ-squared = 16.568, simulated p-value = 0.0013 [57,58]).  While rare, double-headed popliteus muscle prevalence rates range from 0.3-4.4%. Of the 12 individuals who had sex reported, all were male (Table 3), possibly due to sampling bias. Although the sex distribution from [46,53] were not known, the sample from [43] was predominantly male and our sample was predominantly female. Bilateral cases (4) were as common as unilateral ones (5), and the youngest individual known to have a double-headed popliteus was 21. Interestingly, the popliteal artery can pass between the two heads [43,46], although we did not observe this in our individual.  Four studies reported on fabella presence/absence in association with the double-headed popliteus [42,43,46], and fabellae were present in 10/15 of the cases. Given lack of data, we cannot conclude whether double-headed popliteus muscles are more/less common when fabellae are present, but these results imply fabellae are more common in knees with double-headed popliteus muscles. Finally, 3 cases also reported a lack of plantaris muscle when the double-headed popliteus was present. | 8 |
| Risk of bias across studies | 22 | Present results of any assessment of risk of bias across studies (see Item 15). | NA |
| Additional analysis | 23 | Give results of additional analyses, if done (e.g., sensitivity or subgroup analyses, meta-regression [see Item 16]). | NA |
| **DISCUSSION** | | |  |
| Summary of evidence | 24 | Summarize the main findings including the strength of evidence for each main outcome; consider their relevance to key groups (e.g., healthcare providers, users, and policy makers).  We combined the data from the systematic review with our results for the results presented below.  Fabellae were present in 63.6% (7/11) of Gruber’s cases. At a similar time, Gruber published a fabella prevalence rate study [55,56], where he found fabella prevalence to be 17.1% (400/2340). Assuming none of these individuals had double-headed popliteal muscles , fabellae are ~3.7 times more common in individuals with double-headed popliteus muscles (χ-squared = 16.568, simulated p-value = 0.0013 [57,58]).  While rare, double-headed popliteus muscle prevalence rates range from 0.3-4.4%. Of the 12 individuals who had sex reported, all were male (Table 3), possibly due to sampling bias. Although the sex distribution from [46,53] were not known, the sample from [43] was predominantly male and our sample was predominantly female. Bilateral cases (4) were as common as unilateral ones (5), and the youngest individual known to have a double-headed popliteus was 21. Interestingly, the popliteal artery can pass between the two heads [43,46], although we did not observe this in our individual.  Four studies reported on fabella presence/absence in association with the double-headed popliteus [42,43,46], and fabellae were present in 10/15 of the cases. Given lack of data, we cannot conclude whether double-headed popliteus muscles are more/less common when fabellae are present, but these results imply fabellae are more common in knees with double-headed popliteus muscles. Finally, 3 cases also reported a lack of plantaris muscle when the double-headed popliteus was present. | 8-9 |
| Limitations | 25 | Discuss limitations at study and outcome level (e.g., risk of bias), and at review-level (e.g., incomplete retrieval of identified research, reporting bias).  Our review revealed 158 unique results, of which 50 records were reviewed (Figure 4). An additional 20 records were identified through bibliographic reviews. Of the 70 results, 24 were screened further. Six records identified through bibliographic review were excluded because we could not locate usable copies: Calori (1866), Bevan (year unknown), Riolan (year unknown) and Fabrice d'Aquapendente (1687) from [46], and Testut (1884) and Nordlund et al., (1877) from [47]. Of the remaining 18 records, 12 were excluded: 7 contained no original data, 2 were about supernumerary muscle bundles of the popliteus/accessory popliteal muscles and not double-headed popliteus muscles [48,49], and 3 were about proximal popliteal attachments but did not encounter any double-headed popliteus muscles [47,50,51]. It is not uncommon for the origin of the popliteus to have two or more attachments, but these are not separate heads [50]. The results from the remaining 6 are presented in Table 3.  The first study reporting on the double-headed popliteus was from 1871 [42]. Like ours, the superior head of their popliteus originated from a large fabella, but unlike ours, the plantaris was missing from this individual (Figure 5). Following the publication of [42], Gruber reviewed his dissection notes and, of the 250 cadavers considered (n = 242 M, 8 F) and found 11 cases of double-headed popliteus muscles, all in men (prevalence = 4.4%). Three of the cases were bilateral and 5 unilateral. Of the unilateral cases, 3 were found in right legs, 2 in left, and most individuals with double-headed popliteus muscles also had plantaris muscles (9/11). Gruber proposed two classes of double-headed popliteus muscles, one in which the two heads were roughly the same size, and one in which the deep, lateral head was bigger. double-headed popliteus identified in this study does not fit in either classification, as the superior, medial head was larger.  The last four studies from the systematic review more-or-less discuss the double-headed popliteus in passing. One study reviewing lower limb muscular anatomy mentioned two men with double-headed popliteus muscles [46]: one man was 70 years old and had fabella(e), and the other was 22 years old and did not. Another study mentioned finding a double-headed popliteus while dissecting 40 knees but gave no further information [52]. Two double-headed popliteus muscles (one per leg) were found during dissection of a 65 year old male [53], and 3 were incidentally found when reviewing 1039 MRI scans of legs looking for triple-headed gastrocnemii [54]. As the last study was not explicitly looking for double-headed popliteus muscles, it is possible their prevalence rate of 0.3% is a minimum prevalence rate. We combined the data from the systematic review with our results for the results presented below.  Fabellae were present in 63.6% (7/11) of Gruber’s cases. At a similar time, Gruber published a fabella prevalence rate study [55,56], where he found fabella prevalence to be 17.1% (400/2340). Assuming none of these individuals had double-headed popliteal muscles , fabellae are ~3.7 times more common in individuals with double-headed popliteus muscles (χ-squared = 16.568, simulated p-value = 0.0013 [57,58]). | 9 |
| Conclusions | 26 | Provide a general interpretation of the results in the context of other evidence, and implications for future research.  The first study reporting on the double-headed popliteus was from 1871 [42]. Like ours, the superior head of their popliteus originated from a large fabella, but unlike ours, the plantaris was missing from this individual (Figure 5). Following the publication of [42], Gruber reviewed his dissection notes and, of the 250 cadavers considered (n = 242 M, 8 F) and found 11 cases of double-headed popliteus muscles, all in men (prevalence = 4.4%). Three of the cases were bilateral and 5 unilateral. Of the unilateral cases, 3 were found in right legs, 2 in left, and most individuals with double-headed popliteus muscles also had plantaris muscles (9/11). Gruber proposed two classes of double-headed popliteus muscles, one in which the two heads were roughly the same size, and one in which the deep, lateral head was bigger. double-headed popliteus identified in this study does not fit in either classification, as the superior, medial head was larger.  The last four studies from the systematic review more-or-less discuss the double-headed popliteus in passing. One study reviewing lower limb muscular anatomy mentioned two men with double-headed popliteus muscles [46]: one man was 70 years old and had fabella(e), and the other was 22 years old and did not. Another study mentioned finding a double-headed popliteus while dissecting 40 knees but gave no further information [52]. Two double-headed popliteus muscles (one per leg) were found during dissection of a 65 year old male [53], and 3 were incidentally found when reviewing 1039 MRI scans of legs looking for triple-headed gastrocnemii [54]. As the last study was not explicitly looking for double-headed popliteus muscles, it is possible their prevalence rate of 0.3% is a minimum prevalence rate. We combined the data from the systematic review with our results for the results presented below.  Fabellae were present in 63.6% (7/11) of Gruber’s cases. At a similar time, Gruber published a fabella prevalence rate study [55,56], where he found fabella prevalence to be 17.1% (400/2340). Assuming none of these individuals had double-headed popliteal muscles , fabellae are ~3.7 times more common in individuals with double-headed popliteus muscles (χ-squared = 16.568, simulated p-value = 0.0013 [57,58]). | 9 |
| **FUNDING** | | |  |
| Funding | 27 | Describe sources of funding for the systematic review and other support (e.g., supply of data); role of funders for the systematic review. | NA |

*From:*  Moher D, Liberati A, Tetzlaff J, Altman DG, The PRISMA Group (2009). Preferred Reporting Items for Systematic Reviews and Meta-Analyses: The PRISMA Statement. PLoS Med 6(7): e1000097. doi:10.1371/journal.pmed1000097

For more information, visit: **www.prisma-statement.org**.

Page 2 of 2
